# Modeling Soil Organic Carbon Changes Using Signal‐To‐Noise Analysis: A Case Study Using European Soil Survey Datasets

**DOI:** 10.1111/gcb.70813

**Published:** 2026-04-10

**Authors:** Xuemeng Tian, Sytze de Bruin, Florian Schneider, Martin Herold, Kirsten de Beurs

**Affiliations:** ^1^ OpenGeoHub Doorwerth the Netherlands; ^2^ Laboratory of Geo‐Information Science and Remote Sensing Wageningen University & Research Wageningen the Netherlands; ^3^ Thünen Institute of Climate‐Smart Agriculture Germany; ^4^ Helmholtz GFZ German Research Centre for Geosciences, Remote Sensing and Geoinformatics Potsdam Germany

**Keywords:** change detection, digital soil mapping, machine learning, signal‐to‐noise ratio, soil organic carbon

## Abstract

Soil organic carbon (SOC) is a key indicator of soil health and a crucial component of climate mitigation, making its reliable monitoring increasingly important. While Digital Soil Mapping (DSM) based on Machine Learning and Earth Observation (EO) data enables the generation of time series of spatially explicit SOC predictions, detecting temporal changes from these model predictions remains challenging due to the relatively large associated uncertainties. Although prediction uncertainties are now commonly reported, few studies have explicitly accounted for them when assessing SOC change. This study introduces a model‐based signal‐to‐noise ratio (SNR) framework to assess the detectability of SOC change using both the *state‐first* approach—modeling SOC states at each time point and then deriving change—and the *change‐first* approach—modeling SOC change directly from repeated measurements. SNR is defined as the ratio of predicted SOC (concentration, g/kg) change to its modeled uncertainty, enabling evaluation of change‐model reliability at pixel levels. Applied to repeated SOC observations from the pan‐European Land Use and Coverage Area Frame Survey, this framework assesses the reliability of SOC change modeling across multiple land‐cover types using Random Forest and Quantile Regression Forests. At the site level, prediction accuracy was poor and SNR values were consistently low. An illustrative aggregation analysis showed that spatial averaging improved SNR, supporting SOC change assessments at broader scales. However, further work is needed to incorporate land use and management information and to systematically examine how different aggregation schemes affect the results in various contexts, ensuring that aggregated outcomes remain meaningful and policy‐relevant. As an internal metric based on model predictions and their estimated uncertainty, SNR provides a practical diagnostic of change‐model confidence, especially when repeated ground‐truth SOC measurements are not available. We advocate for routine SNR reporting to enhance the transparency and credibility of DSM‐based SOC change monitoring.

## Graphical abstract

This conceptual illustration underpins the Signal‐to‐Noise Ratio (SNR) framework proposed in the study to assess SOC change detectability using repeated Soil Organic Carbon (SOC) observations, Machine Learning and Earth Observation data. Using a simple simulated time series, the figure summarizes the two modelling approaches evaluated in this study: State‐first approach: Models are trained to predict SOC states at each time step. SOC change is then derived by differencing the predicted states, and state‐level uncertainties are propagated to obtain the uncertainty of the derived change. Change‐first approach: SOC change between measurements is first computed, and ML models are trained to predict the change directly along with its associated uncertainty. By treating the magnitude of the estimated change as the signal and the corresponding uncertainty as the noise, SNR can be computed for both approaches. SNR indicates how confidently SOC change can be detected: high SNR reflects a reliable change signal, whereas low SNR indicates that uncertainty overwhelms the predicted change.

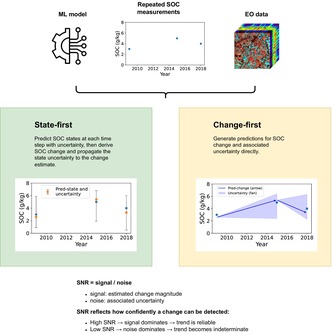

## 1 Introduction

Soil organic carbon (SOC) is widely recognized as a key indicator of soil health and a critical component of climate mitigation, owing to its role in sequestering atmospheric carbon (Lal 2004; Lehmann et al. 2020; Stockmann et al. 2013; Don et al. 2024). Reliable information on SOC dynamics is therefore increasingly sought by scientists, policymakers, and land managers, particularly for monitoring, reporting, and verification (MRV) purposes (Lehmann et al. 2020; Smith et al. 2020).

To meet this growing need for reliable SOC information, digital soil mapping (DSM) has gained momentum in recent years, particularly through approaches combining machine learning (ML) and satellite‐based Earth observation (EO) data (Wadoux et al. 2020). ML captures complex, nonlinear relationships among predictors, while EO provides consistent, high‐resolution, and regularly updated surface information. Together with the expansion of soil survey databases, these advances have driven a surge in spatiotemporal SOC mapping (Hengl et al. 2018; Wadoux et al. 2020). Notable examples include the biannual Worldsoils global maps (van Wesemael et al. 2024), the SoilHealthDataCube for Europe (Tian, de Bruin, et al. 2025), and national‐scale time series for Argentina, the Netherlands, South Africa, Hungary, and others (Heuvelink et al. 2021; Helfenstein et al. 2024; Venter et al. 2021; Szatmári, Laborczi, et al. 2024). Beyond reconstructing past SOC dynamics, several studies have also extended similar approaches to project future trends (Yigini and Panagos 2016; Wang et al. 2022; Zhang et al. 2023; Soils Revealed 2025). From such SOC maps, SOC change is derived by first predicting SOC levels at each time step and then calculating their difference or fitting a trend line (Chen et al. 2019; Yang et al. 2022; Li et al. 2022; Helfenstein et al. 2024; Szatmári, Pásztor, et al. 2024; Meng et al. 2024), an approach here referred to as the *state‐first* strategy.

In contrast, the *change‐first* approach derives SOC changes directly from repeated observations and models those changes as the target variable. By focusing on change rather than absolute states, this strategy can mitigate correlated noise between time steps (De Rosa et al. 2024). It remains less common due to the scarcity of long‐term, repeated SOC measurements. Practical mapping applications are still rare, with De Rosa et al. (2024)'s analysis of repeated Land Use and Coverage Area Frame Survey (LUCAS, Orgiazzi et al. 2018) topsoil samples being one of the few examples.

Uncertainty in SOC prediction can arise from multiple sources, including soil sampling variability, EO data quality, and model assumptions (Heuvelink et al. 2021; Zhang, Huang, and Yang 2024; Even et al. 2025). As a result, accounting for prediction uncertainty has become an integral part of DSM (G. B. Heuvelink 2014, 2018). Various methods have been developed to quantify model uncertainty, among which quantile regression forests (QRF) are widely applied (Vaysse and Lagacherie 2017; Wadoux et al. 2020). Consequently, most recent DSM products now report uncertainty alongside predicted values, typically as prediction intervals derived from model distributions (G. B. Heuvelink 2014; Kasraei et al. 2021; Schmidinger and Heuvelink 2023).

Although uncertainty is particularly relevant when expected SOC changes are small relative to prediction uncertainty (Poeplau and Don 2013; Paustian et al. 2019), few studies have quantitatively tested whether modeled SOC changes exceed their associated uncertainty. In the *state‐first* approach, uncertainty is often visualized through prediction intervals alongside SOC time series (Tian, de Bruin, et al. 2025), which provides useful context but remains qualitative. A rare quantitative example is Szatmári, Pásztor, et al. (2024), who considered uncertainty in SOC change but focused only on trend direction (positive vs. negative). For the *change‐first* approach, applications are even rarer; in De Rosa et al. (2024)'s study, uncertainty was shown alongside predicted SOC change, but only at an aggregated longitudinal level.

To address this gap, we introduce a model‐based signal‐to‐noise ratio (SNR) framework to assess whether predicted SOC changes are distinguishable from model uncertainty. SNR is defined as the ratio between the magnitude of the predicted SOC change (the signal) and its modeled prediction uncertainty (the noise). A high SNR (SNR > 1) indicates that the modeled change exceeds its uncertainty, providing a straightforward internal measure of model confidence—particularly valuable when independent repeated soil samples for external validation are scarce.

We demonstrate the approach with repeated SOC measurements from the pan‐European LUCAS topsoil (0–20 cm) dataset, which enables evaluation across diverse land‐cover types under both the *state‐first* and *change‐first* approaches. Although implemented at the pixel level, the SNR framework can also be applied to aggregated spatial units (e.g., administrative boundaries or regular grids), which is particularly relevant for MRV applications at different spatial levels. Spatial aggregation further improves SNR by dampening localized noise (Wadoux and Heuvelink 2023; Szatmári, Pásztor, et al. 2024; Tian, de Bruin, et al. 2025), a point we demonstrate through case studies in this paper. Although SOC stock (e.g., Mg/ha) is more relevant for climate change mitigation, repeated measurements of bulk density, coarse fragments and depth to bedrock are even scarcer than those of SOC concentration (e.g., g/kg). Hence, this study focuses on SOC concentration, with “SOC” hereafter referring to concentration. While SOC concentration is used in this study due to current data limitations, the proposed SNR framework can, in principle, be extended to SOC stock when the necessary auxiliary data become available.

Through this SNR‐based evaluation, we address the following research questions, which together aim to assess the reliability and detectability of SOC change modeling across spatial scales:

- **RQ1**: How do SOC change prediction capabilities across Europe compare to those reported for the Netherlands (Helfenstein et al. 2024) and German croplands (Broeg et al. 2024)?
- **RQ2**: How do the *change‐first* and *state‐first* strategies differ in predictive accuracy and SNR when modeling SOC change?
- **RQ3**: Which factors influence SNR in SOC change modeling, such as land cover, monitoring duration, sampling frequency, and spatial scales of interest?
- **RQ4**: How does SNR relate to prediction accuracy in SOC change modeling?

## 2 Materials and Methods

### 2.1 Definitions and Notation for SOC and Its Change

We denote the measured SOC (g/kg) at location s and time $t_{i}$ as $c_{s,t_{i}}$. SOC change can be described in two main ways: (i) as the net difference between paired observations over a defined interval ($\delta_{s,t_{1}\to t_{2}}$; g/kg) (Lettens et al. 2005; Poeplau and Don 2013; Harbo et al. 2023), and (ii) as a temporal trend estimated through regression models that relate SOC to time. In the absence of detailed knowledge about temporal SOC dynamics, it is commonly assumed that SOC changes linearly with time, and linear models are fitted across multiple time points. The resulting slope parameters, denoted $\beta_{s,t_{1}\to t_{n}}$ (g/kg/year) (Bellamy et al. 2005; Harbo et al. 2025) represent the average rate of SOC change over the time window $t_{1}\to t_{n}$ for spatial unit s. All SOC change metrics are derived from repeated measurements at the same location ($c_{s,t_{i}}$).

For simplicity, spatial and temporal indices (e.g., s, t) are omitted throughout the manuscript unless explicitly required. Accordingly, SOC measurements and their derived metrics are denoted as c, δ, and β, representing the observed SOC concentration, net change, and temporal trend, respectively. Their corresponding model‐predicted values are denoted as $\hat{c}$, $\hat{\delta }$, and $\hat{\beta }$.

### 2.2 Data Preparation

**FIGURE 1:**
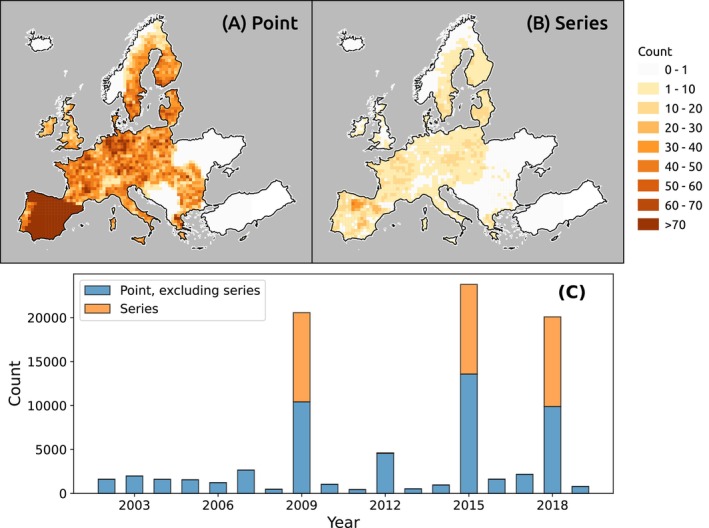
Spatial density of all point measurements (A) and filtered time‐series observations (B) aggregated to 50 km grid cells across Europe, together with their temporal distribution across survey years (C).

In this study, we primarily used soil data from the LUCAS soil database, supplemented with three national soil datasets. Two of these are from Spain: Parcelas COS (top 30 cm depth) and Parcelas INES (top 10 cm) (Serrano et al. 2022). The third dataset, BZE‐LW, is from Germany and represents the core dataset of the first German agricultural soil inventory (Poeplau et al. 2020), containing measurements across multiple soil depths. As the only database providing repeated topsoil samples (0–20 cm) with pan‐European coverage, LUCAS served as the reference dataset and foundation of this study. The analysis focused on topsoil samples within the 0–20 cm depth range. For sites with measurements at multiple depths, SOC values were interpolated to a standardized depth of 10 cm—representing the midpoint of the target layer. To preserve the shape of data and avoid overshooting, we used the Piecewise Cubic Hermite Interpolating Polynomial method (PCHIP) (Fritsch and Butland 1984), which achieved a high coefficient of determination (0.8) in reconstructing SOC values at missing depths in an internal accuracy assessment (Supplementary Notebook 02e). The final harmonized dataset includes 87,684 c observations, from all over Europe (Figure 1A), covering year 2002 to 2019 (Figure 1C). Summary statistics are provided in Table 1.

**TABLE 1:** Summary statistics of point SOC (g/kg) per dataset.

Source	Year	*N* sites	*N* samples	Mean	Median	SD
LUCAS	2009, 2012, 2015, 2018	27,737	62,437	45.98	20.70	82.03
Parcelas INES	2002–2018	21,502	21,502	36.63	23.53	38.67
BZE‐LW	2011–2018	2953	2953	30.07	18.37	49.22
Parcelas COS	2019–2020	789	789	40.78	27.17	34.64

Among the LUCAS observations, a subset includes repeated measurements taken at the same locations over three sampling rounds: 2009/2012, 2015, and 2018, enabling the analysis of SOC dynamics over time. To ensure data consistency and quality, a filtering procedure was applied to keep only consistent SOC time series. The following criteria were used: (1) each time series must contain at least three observations, representing the longest possible duration; (2) the maximum spatial distance between repeated measurements must not exceed 30 m, a practical threshold aligning with the resolution of our previous SOC maps (Tian, de Bruin, et al. 2025), under the simplifying assumption that points within 30 m fall within the same map pixel; and (3) the standard deviation of the carbon‐to‐nitrogen (C/N) ratio across the time series must be less than 3.8, based on the observed bimodal distribution of C/N differences, where 3.8 marks a local minimum between two peaks (see Supplementary Notebook 02b for details; Ziche et al. 2022). This filtering yielded 10,204 SOC time series from the LUCAS dataset, covering most of Europe (Figure 1B), with observations primarily from the years 2009, 2015, and 2018 (Figure 1C). A small subset of time series includes measurements from 2009, 2012, and 2018, though this group is not clearly visible due to its limited size.

The change (δ) was calculated for all valid pairs within each time series, including step‐to‐step (e.g., $t_{1}\to t_{2}$, $t_{2}\to t_{3}$) and longer‐interval (e.g., $t_{1}\to t_{3}$) differences. This resulted in a total of 30,612 δ values across the dataset. We estimated the rate of change at each location using the Theil–Sen estimator—a non‐parametric, robust linear regression method well‐suited for small sample sizes and resistant to outliers (Theil 1950; Sen 1968). Slopes were derived from the full SOC time series at each site, yielding 10,204 β values. This was implemented using TheilSenRegressor from sklearn.linear_model.

An independent test set of 1800 SOC time series (about 20% of the dataset, corresponding to 5400 point observations and 5400 time‐step pairs) was randomly selected from the filtered LUCAS dataset. This size was chosen to ensure a sufficiently large and diverse test set for evaluation, while leaving enough data for training and exploration. This test set was used for evaluating prediction accuracy, assessing uncertainty estimates, and conducting SNR analysis.

### 2.3 Covariates

A wide range of covariates (also referred to as *predictors* or *features*) was included to represent environmental factors influencing soil formation, as described by the SCORPAN model. Specifically, SCORPAN refers to s: soil properties; c: climate; o: organisms (vegetation); r: topography; p: parent material (lithology); a: age; and n: spatial position (McBratney et al. 2003). Topographic variables (Ho et al. 2025) and parent lithology (Isik et al. 2024) were included to represent terrain‐driven controls on hydrology, erosion, and soil formation processes. Plant functional type (Harper et al. 2023) and vegetation cover fraction (Sun et al. 2023) maps were used to characterize vegetation composition and cover, which are directly related to carbon inputs to the soil (Smith 2008). Climate variables include precipitation (Karger et al. 2021), temperature (Wan 2006), and water vapor (Lyapustin and Wang 2018), as well as bioclimatic variables (Karger et al. 2017) that represent biologically meaningful climate constraints relevant to vegetation growth and soil carbon processes. Thematic peatland (Widyastuti et al. 2024) and cropland (Potapov et al. 2022) datasets were included to represent persistently waterlogged peat soils with high SOC contents and intensively managed croplands with direct human intervention. Soil moisture (Bauer‐Marschallinger et al. 2018) was included to indicate water availability and aeration conditions that regulate organic matter decomposition and SOC stabilization. In addition, less‐processed remote sensing signals were incorporated, including Landsat reflectance and derived spectral indices (Tian, Consoli, et al. 2025) and bare‐soil composites from Sentinel imagery (Rogge et al. 2018). Two Synthetic Aperture Radar (SAR) backscatter signals at different wavelengths (Shimada and Ohtaki 2010; Wagner et al. 2021) were also included. These signals are sensitive to surface properties and vegetation structure and have been shown to be informative for modeling SOC in superficial soil layers (Baghdadi et al. 2008).

Some covariates were available as time series, providing multiple observations for the same location over time, including vegetation‐related variables, climate variables, cropland information, and soil moisture. Other covariates provided only a single value per location, including static variables or long‐term summaries. Static layers include slowly varying properties such as topography and lithology, as well as variables treated as static as a pragmatic compromise given data availability and consistency at the continental scale, including Synthetic Aperture Radar (SAR) backscatter and peatland cover. Long‐term summaries were derived from multi‐year time series for selected covariates and include statistical descriptors such as the mean, percentiles, and standard deviation, resulting in a single value per location. In this study, “long‐term” refers to the period from 2000 to 2022, corresponding to the time span over which most dynamic covariates are consistently available.

The resulting initial feature pool consisted of 582 individual covariates, organized into 15 covariate groups, which in turn represent seven broader SCORPAN classes (Table 2). The full list of covariates is not included in the main text but is available in the Supporting Information at our Supplemetary Repository. Various preprocessing steps were applied to the original datasets, including temporal aggregation, gap‐filling of missing values, and reprojection to ensure consistent coordinate reference systems, spatial coverage, and temporal alignment across all layers.

**TABLE 2:** Covariate groups used in this study.

Class	Covariate group	Source	Temporal resolution	Spatial resolution
Topography	Digital terrain model and derived land surface parameters	Ho et al. (2025)	Static	, 60, 120, 240, 480, and 960 m
Lithology	Lithology type probability	Isik et al. (2024)	Static	m
Vegetation	Plant functional type	Harper et al. (2023)	Annual	m
Vegetation	Vegetation cover fraction	Sun et al. (2023)	Annual	m
Climate	Precipitation	Karger et al. (2021)	Annual	km
Climate	Land surface temperature	Wan (2006)	Annual	km
Climate	Water vapor	Lyapustin and Wang (2018)	Annual	km
Climate	Bioclimate	Karger et al. (2017)	Long‐term	km
Imagery	Landsat spectral index	Tian, Consoli, et al. (2025)	Annual, long‐term	m
Imagery	Bare‐soil composite	Rogge et al. (2018)	Long‐term	m
Imagery	Sentinel‐1 SAR backscatter	Wagner et al. (2021)	Static	m
Imagery	PALSAR backscatter	Shimada and Ohtaki (2010)	Static	m
Land cover	Peatland	Widyastuti et al. (2024)	Static	km
Land cover	Cropland	Potapov et al. (2022)	Annual	m
Soil	Soil moisture	Bauer‐Marschallinger et al. (2018)	Annual	km

*Note:* Note that each entry represents a group of covariate layers, which may include multiple datasets with different temporal and spatial resolutions.

These covariate layers were overlaid with c observations based on spatial coordinates (latitude and longitude) and temporal information (year) to construct the training matrix for the $RF_{c}$. To reduce dimensionality and improve interpretability, we applied the Repeated Subsampling‐Based Cumulative Feature Importance (RSCFI) method (Tian, de Bruin, et al. 2025). This approach, conceptually similar to recursive feature elimination with cross‐validation, iteratively removes features with low cumulative importance scores, thereby retaining only the most relevant predictors while balancing model performance and computational efficiency. Details of the RSCFI implementation are provided in Supplementary Notebook 07a.

The covariates identified as important by $RF_{c}$ were then organized to reflect environmental changes over time, supporting the construction of training matrix for $RF_{\delta }$ and $RF_{\beta }$. Following Yang et al. (2022), we applied time‐series feature engineering to convert state‐based covariates into change‐based representations. Each covariate time series was partitioned into three periods: (i) the change interval (between start and end years), (ii) the years preceding the change (from 2000—the earliest common year—up to the start year), and (iii) the full period from 2000 to the end year. From each period, we derived summary statistics of feature values—mean, standard deviation, and linear slope (as a proxy for trend)—yielding a structured set of dynamic predictors aligned with the temporal spans of δ and β. These were then used to train $RF_{\delta }$ and $RF_{\beta }$.

### 2.4 SOC Model Approaches

All models are implemented using RF and QRF, two widely used methods in DSM for SOC prediction and uncertainty quantification (Hengl et al. 2018; Wadoux et al. 2020). We used a modified version of the RF algorithm to capture full prediction distributions by storing values from all valid nodes across trees. This mimics the behavior of QRF, enabling simultaneous estimation of the target variable (via the mean) and associated uncertainty (via the standard deviation). This was implemented by modifying the RandomForestRegressor in scikit‐learn to keep node‐level outputs. Three separate RF models were constructed using the same workflow, including feature selection, hyperparameter tuning, and model fitting, to predict c, δ and β, respectively.

- *state‐first*: This approach relies on a single model, $RF_{c}$, to generate spatial–temporal $\hat{c}$, from which $\hat{\delta }$ and $\hat{\beta }$ are subsequently derived.
  – **Model of spatial–temporal point SOC values (** $RF_{c}$ **)**: The training matrix was constructed by aligning SOC observations with the corresponding environmental features, drawn from 15 covariate groups (Table 2), based on spatial coordinates (latitude and longitude) and temporal information (year).
- *change‐first*: In this approach, the target variable directly corresponds to the modeled output. Temporal dynamics are incorporated through feature engineering of covariates, aiming to reflect underlying processes rather than static states (see next section for details).
  – **Model of paired SOC change (** $RF_{\delta }$ **)**: The training matrix was constructed by matching δ values with features that reflect environmental changes over the corresponding time period at the same location.
  – **Model of SOC trend slope (** $RF_{\beta }$ **)**: As with the $RF_{\delta }$, covariates were engineered to reflect environmental processes across the time span of each series.

Performance metrics—including mean absolute error (MAE), bias, and concordance correlation coefficient (CCC)—were calculated on an independent test set to evaluate the prediction accuracy of the three models: $RF_{c}$, $RF_{\delta }$, and $RF_{\beta }$, used under both the *state‐first* and *change‐first* approaches. This resulted in five prediction scenarios (Table 3).

**TABLE 3:** Overview of RF models used in the *state‐first* and *change‐first* approaches and their corresponding target variables.

Approach	Model	Target	Predicted
*State‐first*	$RF_{c}$	c	$\hat{c}$
		δ	$\hat{\delta }$ derived as temporal differences of $\hat{c}$
		β	$\hat{\beta }$ derived as temporal slopes fitted to $\hat{c}$
*Change‐first*	$RF_{\delta }$	δ	$\hat{\delta }$
	$RF_{\beta }$	β	$\hat{\beta }$

We used the standard deviation of RF node outputs as a proxy for prediction noise in the SNR calculation—an approach valid only if this spread reliably reflects uncertainty. Therefore, the models were also evaluated for the validity of their uncertainty estimates using two metrics: the Prediction Interval Coverage Probability (PICP), which measures the proportion of observed values falling within the predicted intervals; and the Quantile Coverage Probability (QCP), which assesses the symmetry of the uncertainty distribution by evaluating single‐quantile predictions. Accuracy plots of PICP and QCP—scatter plots comparing observed and expected coverage rates across a range of expected prediction intervals—were used to visualize and evaluate local uncertainty estimation performance, as recommended by Schmidinger and Heuvelink (2023).

### 2.5 SNR Analysis

The SNR was defined as the ratio between the model's predicted value and its associated prediction uncertainty. SNR thus quantifies the extent to which a modeled change exceeds its own uncertainty, providing an internal diagnostic of model confidence. Its specific computation varies by model, target variable, and approach, as shown in Table 4, where the numerator represents the *signal*—the absolute magnitude of the predicted target ($|\hat{c}|$, $|\hat{\delta }|$, or $|\hat{\beta }|$). Although the calculation of SNR varies across models, all rely on the prediction distribution—that is, the set of terminal node values from the QRF. This conditional distribution is assumed to approximate model uncertainty within the local feature space.

**TABLE 4:** SNR calculation formulas for each model and target variable.

Target	Model		
	$RF_{c}$	$RF_{\delta }$	$RF_{\beta }$
	$R=\left\{{\hat{c}}^{\left(1\right)},{\hat{c}}^{\left(2\right)},\ldots ,{\hat{c}}^{\left(n\right)}\right\}$	$R=\left\{{\hat{\delta }}^{\left(1\right)},{\hat{\delta }}^{\left(2\right)},\ldots ,{\hat{\delta }}^{\left(n\right)}\right\}$	$R=\left\{{\hat{\beta }}^{\left(1\right)},{\hat{\beta }}^{\left(2\right)},\ldots ,{\hat{\beta }}^{\left(n\right)}\right\}$
c	$\frac{\hat{c}}{\mathrm{std}\left(R\right)}$	—	—
δ	$\frac{|\delta \left({\hat{c}}_{t_{0}},{\hat{c}}_{t_{1}}\right)|}{\sqrt{\mathrm{std}{\left(R_{t_{0}}\right)}^{2}+\mathrm{std}{\left(R_{t_{1}}\right)}^{2}}}$ *	$\frac{|\hat{\delta }|}{\mathrm{std}\left(R\right)}$ ^#^	—
β	$\frac{|\beta \left({\hat{c}}_{t_{0}},{\hat{c}}_{t_{1}},{\hat{c}}_{t_{2}}\right)|}{\mathrm{std}\left(\beta \left(R_{t_{0}},R_{t_{1}},R_{t_{2}}\right)\right)}$ *	—	$\frac{|\hat{\beta }|}{\mathrm{std}\left(R\right)}$ ^#^

*Note:* R denotes the prediction distribution, that is, all prediction instances for a target variable obtained from the full valid terminal node values of the RF.

* Indicates the *state‐first* approach.

^#^ Indicates the *change‐first* approach.

In the *change‐first* approach, noise is quantified as the standard deviation of this prediction distribution. While this is similar to QRF, which uses the same prediction set to estimate conditional quantiles (Meinshausen 2006), our method focuses on capturing the uncertainty level via spread. This procedure is applied consistently across the three direct models ($RF_{c}$, $RF_{\delta }$, and $RF_{\beta }$), corresponding to the diagonal entries in Table 4.

For the *state‐first* approach, the $RF_{c}$ first predicts $\hat{c}$ values, from which both $\hat{\delta }$ and $\hat{\beta }$ are derived (Table 3). To estimate noise in $\hat{\delta }$, we assumed the two $\hat{c}$ estimates to be independent, as RF models treat samples independently. Under this assumption, the noise for $\hat{\delta }$ is computed as the square root of the sum of variances from both time steps. To estimate noise for $\hat{\beta }$, we implemented a Monte Carlo simulation. For each iteration, $\hat{c}$ values were randomly sampled from the full prediction distribution (i.e., all valid leaf node outputs) at each time step, and a linear regression was fitted to estimate the corresponding slope. This process was repeated 200 times, generating a distribution of $\hat{\beta }$ values. The standard deviation of this distribution was used as the noise estimate for $\hat{\beta }$.

To explore the relationship between internal model confidence (SNR) and actual predictive accuracy, we compared SNR with relative absolute error (RAE) for each $\hat{\delta }$ and $\hat{\beta }$ in the validation dataset. RAE was used to evaluate prediction accuracy across all target variables (c, δ, and β). It is defined as the absolute difference between the predicted and observed values, normalized by the observed value.

### 2.6 Spatial Aggregation

While spatial averages can be calculated by averaging predictions over the area of interest (AOI), quantifying the corresponding uncertainty is less straightforward—particularly owing to the spatial dependence of prediction errors. In this study, we adopted an approach similar to that used by Araza et al. (2022) and Wadoux and Heuvelink (2023), which estimates the uncertainty of spatial aggregates using model‐based point‐support uncertainty‐prediction standard deviation, in our case, derived from discretization points within the AOI. This method avoids the numerical complexity of traditional statistical techniques, making it especially suitable for large‐scale applications. Specifically, it is implemented as follows:

(1) $${\mathrm{sd}}_{\mathrm{AOI}}=\sqrt{\frac{1}{{\left|B\right|}^{2}}\int_{s\in B}\int_{u\in B}\mathrm{sd}\left(s\right)\cdot \mathrm{sd}\left(u\right)\cdot \rho \left(s-u\right)\;\mathrm{ds}\;\mathrm{du}}$$

The B in Equation (1) represents the number of discretization points, generated from the AOI; $\mathrm{sd}\left(s\right)$ and $\mathrm{sd}\left(u\right)$ denote the standard deviations of the point support prediction residuals at locations s and u, assuming the prediction errors are proportional to the prediction uncertainty (standard deviation of prediction distribution from QRF) at point support. The $\rho \left(s-u\right)$ represents the correlation function of the standardized prediction error at the separation distance between s and u. Standardized prediction errors and corresponding uncertainty were derived through cross‐validation, where predictions were made on samples excluded from model training. Errors were calculated as the difference between observed and predicted values, then standardized by dividing by the prediction uncertainty. We assumed second‐order stationarity of the standardized errors—that is, their spatial correlation depends only on the Euclidean distance between locations. The correlation structure was estimated by fitting a variogram $\gamma \left(h\right)$ using:

(2) $$\rho \left(h\right)=\frac{\text{sill}-\gamma \left(h\right)}{\text{sill}},$$

where sill denotes the fitted sill of the variogram. To assess the effect of spatial aggregation on SNR, SOC prediction maps at 1 km^2^ resolution were first generated, which served as the basis for subsequent aggregation. We considered four levels of spatial support, a concept from geostatistics describing the area over which a variable is aggregated: 2.5, 10, 40, and 200 km^2^. These scales, spanning fine to coarse resolutions, were chosen to demonstrate the effect of spatial aggregation on SNR and are not intended as prescriptive values. The analysis focused on Germany and Spain, where additional data is available. Each country was divided into square grid units corresponding to the four support sizes. Within each grid unit, map pixels were used as discretization points for aggregation, and B denotes the number of pixels contained in each grid unit at the respective spatial support.

Spatial aggregation was applied only to the $RF_{c}$ model under the *state‐first* approach. This restriction was mainly due to the limited density of available SOC time series measurements, which must be sufficient to fit standardized error variograms for aggregated uncertainty estimation. Moreover, as *state‐first* approach remains the standard in SOC change modeling, results are broadly applicable to current SOC time series products.

## 3 Results

### 3.1 Prediction Accuracy and Uncertainty Estimation Evaluation

**FIGURE 2:**
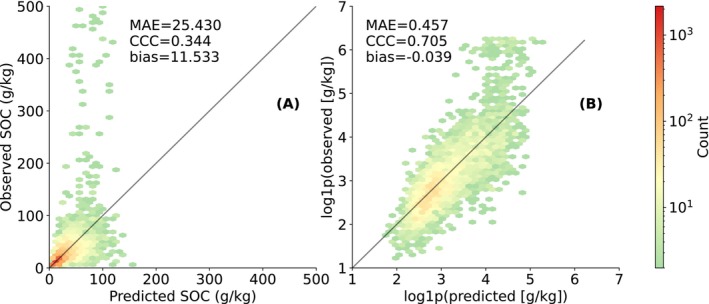
Prediction accuracy of c using $RF_{c}$. Panel (A) shows the density plot of observed versus predicted values on the original scale (g/kg), and panel (B) shows the same results with log‐transformed values (log1p transformed g/kg) for greater detail at lower concentrations. Both panels are based on the same validation dataset. Performance metrics are calculated and presented in each panel.

The prediction accuracy of $\hat{c}$ was first evaluated to justify the use of the *state‐first* approach. $RF_{c}$ achieves a CCC of 0.344 in the original scale (Figure 2A) and 0.705 in the log‐transformed scale (Figure 2B). These values are comparable to those reported in previously published continental‐scale DSM models (Tian, de Bruin, et al. 2025; van Wesemael et al. 2024). While the model shows a slight positive bias—tending to overestimate SOC—it consistently underestimates the highest observed SOC values.

**FIGURE 3:**
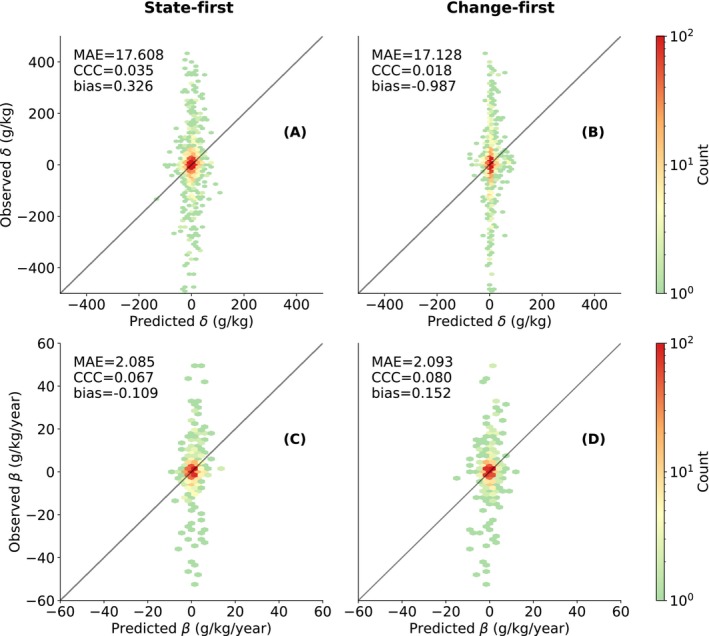
Prediction accuracy of δ and β using two approaches: *State‐first* based on predictions from $RF_{c}$ (A and C), and *change‐first* using $RF_{\delta }$ (B) and $RF_{\beta }$ (D). Performance metrics are shown in the upper‐left corner of each subplot.

Figure 3 shows the accuracy of SOC change predictions—$\hat{\delta }$ (top) and $\hat{\beta }$ (bottom)—using both *state‐first* (left) and *change‐first* (right) approaches. Unlike the good performance for $\hat{c}$, $\hat{\delta }$ and $\hat{\beta }$ show poor accuracy across all models and approaches, with all models yielding near‐zero CCC and high MAE relative to the scale of the target variables. For *state‐first* (Figure 3A,C), predictions are weakly correlated with observations. Accuracy does not improve under *change‐first* (Figure 3B,D). Both approaches struggle to reliably capture the correct direction of change. The *state‐first* and *change‐first* approaches yield conflicting biases for both $\hat{\delta }$ and $\hat{\beta }$.

Despite overall poor performance, *state‐first* approach (Figure 3A) shows a wider spread of $\hat{\delta }$ predictions than *change‐first* (Figure 3B), where predictions cluster around zero. This contrast is less pronounced for $\hat{\beta }$ (Figure 3C,D).

**FIGURE 4:**
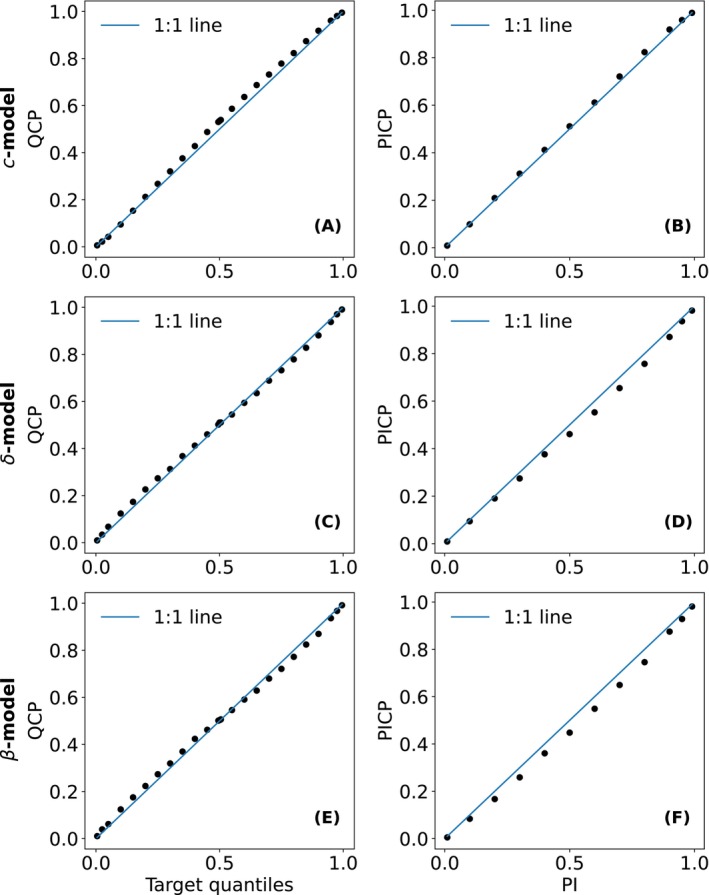
Accuracy plots of QCP (left column, A, C and E) and PICP (right column, B, D and F) used to evaluate the $RF_{c}$ (A and B), $RF_{\delta }$ (C and D), and $RF_{\beta }$ (E and F)—in estimating uncertainty for their respective target variables. All plots present scatter points comparing observed coverage rates on the y‐axis with expected coverage rates on the x‐axis across a range of prediction intervals.

All three models demonstrated reasonable performance in estimating uncertainty when applied to their direct target under the *change‐first* approach (Figure 4). A slight underestimation—indicative of over‐optimism—was observed in the PICP of the $RF_{\delta }$ and $RF_{\beta }$ (Figure 4D,F) when the expected prediction interval was around 50%. When assessing the sharpness of prediction intervals, the results (see Supplementary Notebook 15) show that the uncertainties estimated by $RF_{\delta }$ and $RF_{\beta }$ under the *change‐first* approach are generally narrower but exhibit less variation.

### 3.2 SNR Analysis of SOC and Its Change Modeling

**FIGURE 5:**
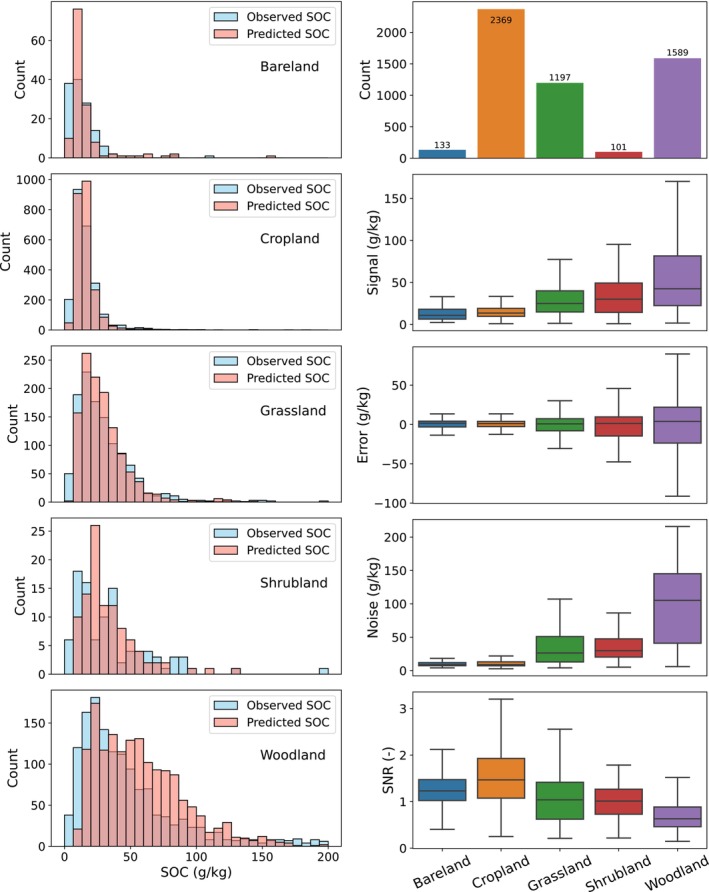
Analysis of the SNR for $\hat{c}$ by $RF_{c}$. Left: Histograms of observed and predicted SOC values across different land‐cover types. Right: Summary statistics of sample count, signal ($\hat{c}$), error, noise, and SNR derived from the independent test set for each land‐cover type. In the right panel, boxes indicate the interquartile range, and whiskers represent the remaining distribution excluding outliers. Note that both plots are based solely on the independent test set.

Model‐based SNR provides an internal diagnostic of SOC change detectability, especially when repeated ground‐truth data are unavailable. The SNR analysis for $\hat{c}$ is presented in Figure 5, stratified by land‐cover type. All Level 1 land cover classes from the LUCAS survey are represented in the study, except for *Wetland and Water Areas* and *Artificial Land*, which were excluded due to insufficient sample sizes (fewer than 10 observations in the test set). As shown in Figure 5, the histograms of observed and predicted SOC generally align for *Bareland*, *Cropland*, *Grassland*, and *Shrubland*, while a clear mismatch is observed for *Woodland*. In *Woodland*, the predicted SOC values are shifted toward higher concentrations and appear compressed, with less extreme values at both ends.

As the independent test set is a random subset of the full dataset, the sample counts reflect the original data distribution, with the majority of observations coming from *Cropland*, followed by *Woodland* and *Grassland*. *Bareland* and *Shrubland* are minimally represented. The signal component increases from *Bareland* to *Woodland*, consistent with increasing vegetation content. Both prediction errors and noise estimates also increase along this gradient. In terms of SNR, *Cropland* shows the highest values, while *Woodland* has the lowest, despite having the second‐highest number of available samples. SNR values are mostly above one for *Bareland* and *Cropland*, close to one for *Grassland* and *Shrubland*, and predominantly below one for *Woodland*.

**FIGURE 6:**
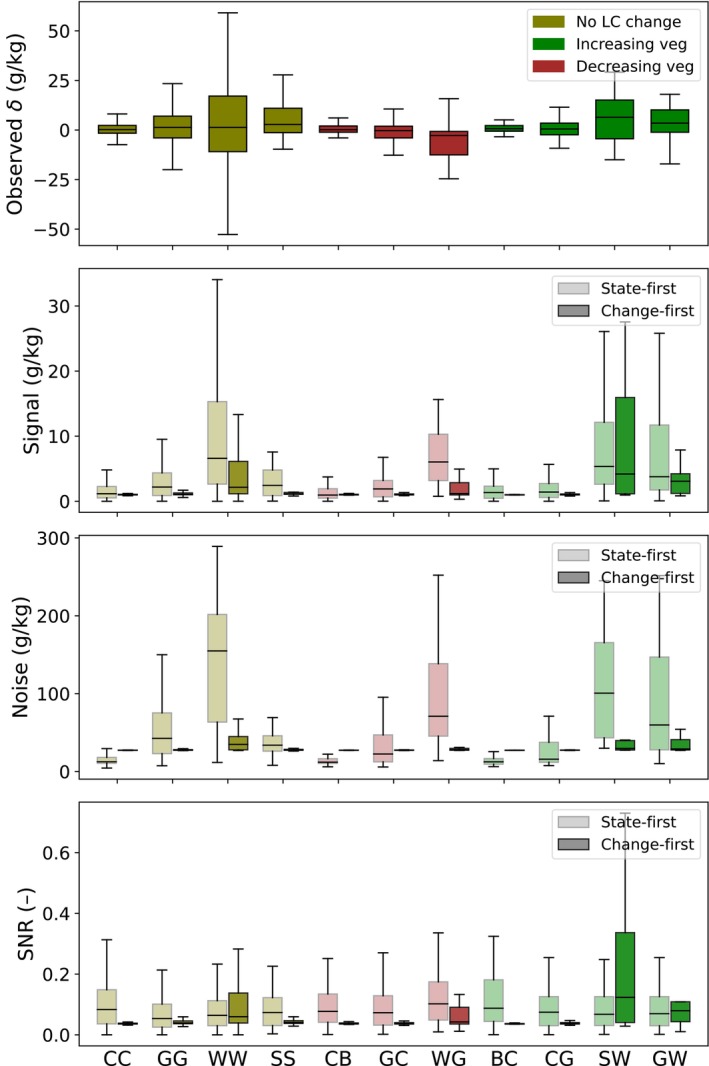
Summary statistics of observed δ, as well as signal, noise, and SNR of $\hat{\delta }$, comparing results from the $RF_{\delta }$ under the *change‐first* approach and the $RF_{c}$ under the *state‐first* approach, grouped by land cover transition pairs. Bars represent median values, and whiskers indicate the 5th to 95th percentile range. Land cover codes are as follows: B = Bareland, C = Cropland, G = Grassland, S = Shrubland, and W = Woodland. For example, “CC” indicates that the location was classified as Cropland in both sampling years. Based on the general increase in vegetation content along the gradient B → C → G → S → W, land cover transitions are grouped into three categories shown in the top panel: No change, increasing vegetation content, and decreasing vegetation content.

Figure 6 presents the SNR of $\hat{\delta }$ (bottom panel) for both the *state‐first* and *change‐first* approaches, along with the corresponding signal, noise, and the distribution of observed δ for reference. Observed δ values tend to be larger and more variable in land‐cover types with higher vegetation. For example, δ is larger when *Woodland* is involved—either when the land cover remained as *Woodland* across both sampling years or transitioned to or from it. In contrast, transitions involving *Cropland* show lower magnitudes and narrower spreads. Although the exact values of δ and $\hat{\delta }$ do not match at the individual prediction level (Figure 3), both the *state‐first* and *change‐first* approaches reproduce the overall trend of δ in $\hat{\delta }$ across land‐cover types, with higher predicted signal magnitudes for transitions involving vegetation‐rich classes.

Overall, the *change‐first* approach yields lower SNR values than the *state‐first* approach, except for transitions involving *Woodland* (e.g., WW, SW, and GW). The variation in SNR across land‐cover types is more pronounced for the *change‐first* approach. This approach reproduces the signal pattern across land‐cover classes—where vegetation‐rich covers correspond to larger signals—while maintaining relatively stable noise estimates. Consequently, transitions involving high‐vegetation land covers show higher SNR values and greater variation across classes. In contrast, under the *state‐first* approach, although the strongest and most variable signals also occur in high‐vegetation classes such as *Woodland*, the noise magnitudes follow similar land‐cover patterns as the signal and δ. As a result, SNR values remain relatively consistent across transitions and generally below 0.4.

**FIGURE 7:**
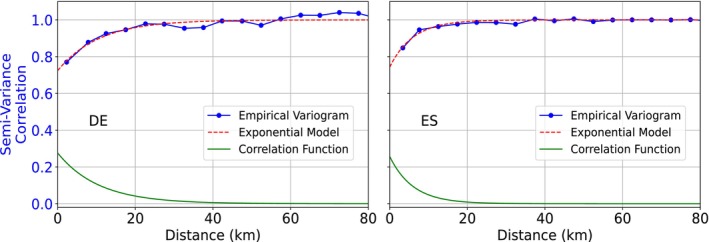
Summary statistics of signal (top panel), noise (middle panel), and SNR (bottom panel) for β, comparing results from the β‐model under the *change‐first* approach and the c‐model under the *state‐first* approach, grouped by land cover time series. Bars represent median values, and whiskers indicate the 5th to 95th percentile range. Land cover codes follow those used in Figure 6, and time series are grouped into three categories: No change, increasing vegetation content, and decreasing vegetation content.

Compared to $\hat{\delta }$, the SNR estimates for $\hat{\beta }$ from both approaches show stronger agreement, with more similar magnitudes and variation across land‐cover types, although the overall SNR patterns for β remain similar to those for δ (Figure 7). Observed β values are higher and more variable for transition types involving vegetation‐rich land covers, a pattern mirrored in the predicted signals from both the *state‐first* and *change‐first* approaches. For the *change‐first* approach, noise estimates remain relatively stable, resulting in larger SNR variations across land‐cover transitions compared with the *state‐first* approach.

For both $\hat{\delta }$ and $\hat{\beta }$, only a weak positive correlation was observed between SNR and RAE (Spearman's rank correlation coefficient was 0.17 in both cases for the *state‐first* approach and close to zero for the *change‐first* approach; see Supplementary Notebook 12). The majority of predictions fall into the “high RAE and low SNR” quadrant, indicating that large prediction errors are generally associated with low detectability of modeled SOC changes.

### 3.3 The Influence of Time Span and Sampling Times

Table 5 shows no substantial differences in the mean values of signal, noise, or SNR for $\hat{\delta }$ across different time intervals, regardless of the approach used. Notably, SNR values based on nine‐year SOC observation pairs are not higher than those derived from three‐year intervals. This is also observed in the δ reported by LUCAS (Supplementary Notebook 04b).

**TABLE 5:** Summary statistics of signal, noise, and SNR for δ across different time intervals, based on predictions from *state‐first* and *change‐first* approach.

Category	ΔT = 3		ΔT = 6		ΔT = 9	
	*State‐first*	*Change‐first*	*State‐first*	*Change‐first*	*State‐first*	*Change‐first*
Count	1811	1811	1800	1800	1789	1789
Signal	5.12	2.47	4.97	3.34	5.56	3.23
Noise	63.09	31.74	64.35	32.21	64.30	32.17
SNR	0.09	0.06	0.09	0.08	0.10	0.08

Compared to δ, which is derived from paired SOC observations between 2009 and 2018, the estimation of β over the same period incorporates an additional intermediate SOC observation. When comparing the SNR of $\hat{\delta }$ and $\hat{\beta }$ across this nine‐year span, $\hat{\beta }$ (0.11 for the *state‐first* and 0.16 for the *change‐first* approach) consistently exhibits slightly higher SNR values than $\hat{\delta }$ (0.10 for the *state‐first* and 0.08 for the *change‐first* approach), regardless of the approach used.

### 3.4 SNR at Larger Spatial Support

**FIGURE 8:**
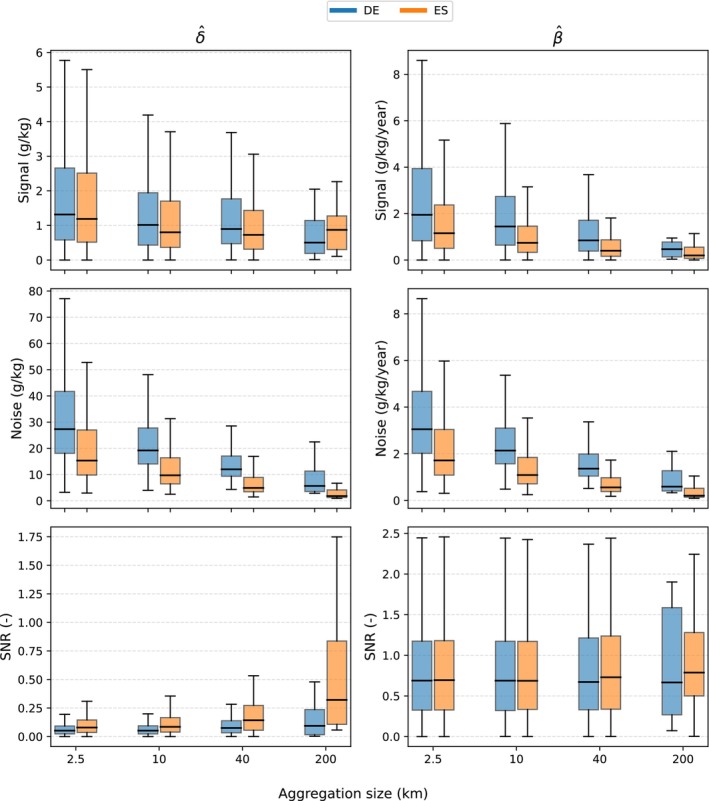
Variograms and corresponding correlation functions of standardized prediction errors for c in Germany (left, nugget: 0.72, range: 10.60) and Spain (right, nugget: 0.74, range: 5.99).

The correlation function of the standardized errors from $\hat{c}$ was estimated using variograms to support the calculation of uncertainty in spatial aggregates. Separate variograms were derived for Germany and Spain (Figure 8). While both exhibit similar nugget values (variance at zero distance; often attributed to measurement error), the variogram for Germany has a noticeably larger spatial range parameter (about 10.60 km vs. 5.99 km for Spain), indicating that prediction errors in Germany remain spatially correlated over longer distances.

**FIGURE 9:**
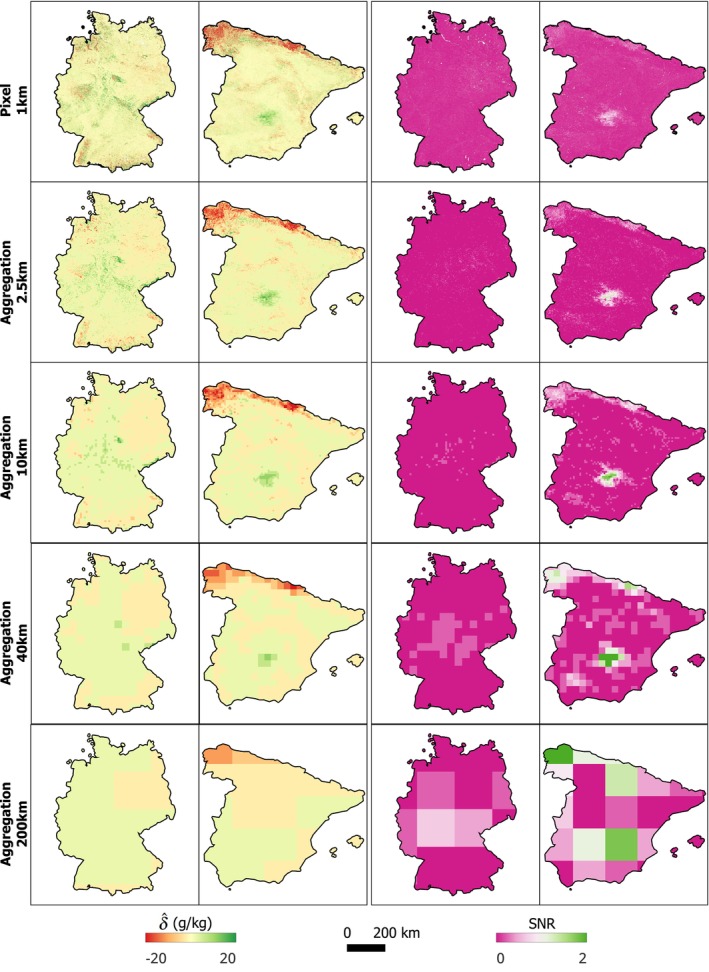
Box plots of signal, noise, and SNR for $\hat{\delta }$ and $\hat{\beta }$ in Germany and Spain for spatial aggregation unit sizes of 2.5 km, 10 km, 40 km and 200 km. Boxes represent the interquartile range, and whiskers indicate the remainder of the distribution. Note the difference in the y‐axes.

Figure 9‐left panel shows that for $\hat{\delta }$, both the signal and the noise generally decline as the spatial aggregation support increases, except for a slight signal increase in Spain between 40 km and 200 km. Overall, the reduction in noise is greater than the reduction in signal, resulting in a steady increase in SNR in both countries. The spread of both signal and noise also narrows with increasing aggregation size, whereas the spread in SNR widens. The SNR increase with spatial aggregation is less pronounced in Germany than in Spain.

For $\hat{\beta }$, both the signal and noise also decrease as the aggregation size increases. However, the rates of decrease are similar, so the SNR improves only marginally. In Germany, the SNR remains relatively constant across all aggregation levels, while in Spain it increases only slightly.

**FIGURE 10:**
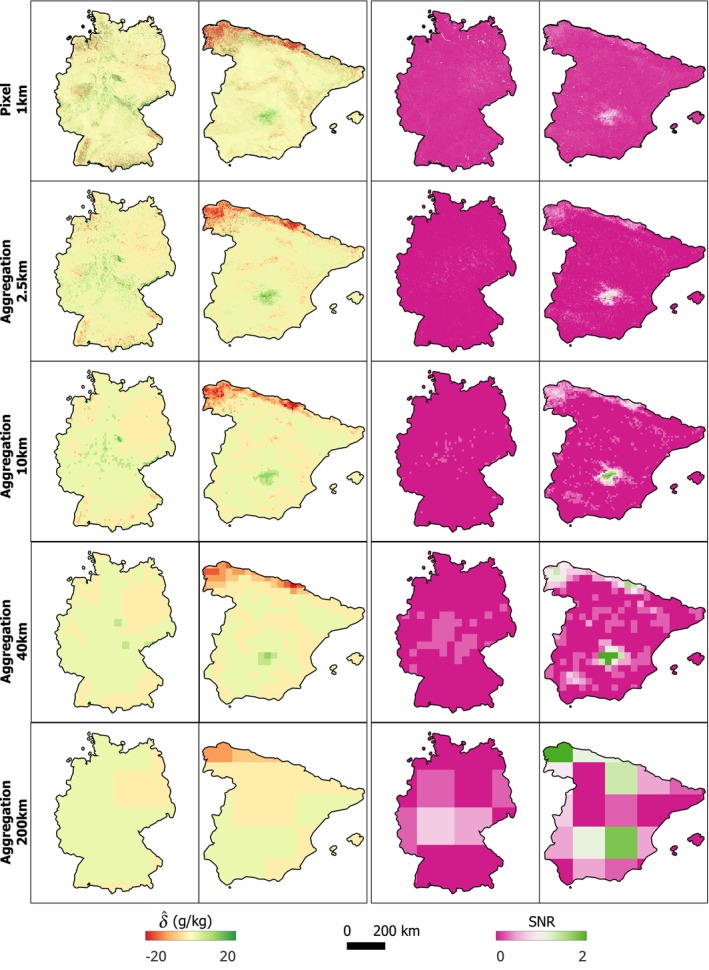
Spatial distribution of $\hat{\delta }$ (left two columns) and corresponding SNR (right two columns) from 2009 to 2018 in Germany and Spain, quantified using the *state‐first* approach. Results are shown across increasing spatial aggregation scales: 1 km pixel‐level predictions, and aggregated units of 2.5 km, 10 km, 40 km and 200 km. Map lines delineate study areas and do not necessarily depict accepted national boundaries.

As the aggregation unit size increases, $\hat{\delta }$ values tend to converge toward zero, yet many grid cells in both countries exhibit increasing SNR values (Figure 10). The spatial distribution of $\hat{\delta }$ and its SNR from 2009 to 2018 indicates that the SNR increase with larger units is more pronounced in Spain, where several grid cells exceed an SNR of one. In contrast, although SNR also increases in Germany, most grid cells remain below one.

## 4 Discussion

### 4.1 Poor Accuracy in Predicting SOC Change

We evaluated two approaches to modeling SOC change—*state‐first* and *change‐first*—with δ and β as target variables. Across all four scenarios, prediction accuracy for SOC change remained low (Figure 3).

Most existing DSM SOC time series adopt the *state‐first* approach (Gasch et al. 2015; Hengl et al. 2017; Poggio et al. 2021; Heuvelink et al. 2021; Li et al. 2022; Helfenstein et al. 2024; van Wesemael et al. 2024; Szatmári, Pásztor, et al. 2024; Tian, de Bruin, et al. 2025), which are often used for change detection and driver analysis. However, consistent with findings from the Netherlands (Helfenstein et al. 2024), the accuracy of SOC change modeling remains poor despite high accuracy in $\hat{c}$.

A key limitation of the *state‐first* approach is that it treats predictions for different time instances as independent variables, ignoring temporal autocorrelation in both SOC and environmental covariates (Poggio et al. 2021; Szatmári, Pásztor, et al. 2024; Tian, de Bruin, et al. 2025). As a critical component of SCORPAN (McBratney et al. 2003), the time factor is therefore not well integrated into current *state‐first* space–time DSM frameworks. Efforts to address this gap include incorporating the age of observations as a covariate (Batjes et al. 2020), using lagged vegetation indices that weight past conditions to reflect delayed SOC responses (Heuvelink et al. 2021), or explicitly incorporating past land use changes in the input covariates (Helfenstein et al. 2024). However, constructing such covariates is often impractical at large scales. Moreover, spatial variability in SOC typically outweighs subtle temporal variation, limiting the benefits of such preparation (Poggio et al. 2021; Tian, de Bruin, et al. 2025). This pattern is also reflected in the exploratory feature contribution analysis (see Supplementary Notebook 14), where static features exert a stronger overall influence on model predictions than temporal variables. As a result, models tend to overfit spatial patterns while underrepresenting temporal dynamics, projecting spatial relationships—such as higher SOC under denser vegetation—onto the temporal dimension, even when actual SOC changes occur more slowly than vegetation.

The *change‐first* approach takes a different strategy by modeling SOC change directly. In theory, this offers two advantages: (1) correlated noise in SOC values may cancel out when computing change, and (2) delayed environmental effects may be better captured by directly linking environmental dynamics to SOC change. However, as shown in Figure 3, SOC change prediction accuracy did not improve as expected. Although the predictive accuracy of $\hat{\beta }$ was slightly higher than that of $\hat{\delta }$, both appeared largely random when validated against repeated measurements, with predictions clustering around zero. This reflects not only the model's limited ability to resolve change, but also the inherent nature of SOC as a slow‐changing property, for which true changes occur over long time scales and are typically small over short intervals (Smith 2004; Poeplau and Don 2013; Gubler et al. 2019). This finding aligns with Broeg et al. (2024), who attempted to predict β using a much longer (35‐year) cropland SOC time series in Bavaria. While their model struggled to accurately estimate β, it succeeded in classifying SOC change direction (increase, decrease, stable). In contrast, with only the nine‐year time span of LUCAS observations, our study shows that $\hat{\beta }$ predictions could not even resolve the direction of change.

### 4.2 SNR as a Predicted SOC Change Assessment Metric

Several studies have applied the concept of SNR to describe challenges in detecting SOC change. For instance, Stevens et al. (2008), Croft et al. (2012) reported low SNR in EO reflectance data caused by illumination, terrain, and atmospheric effects, while Paustian et al. (2019) highlighted that SOC changes are often masked by natural spatial variability. Broeg et al. (2024) quantified SNR by defining the signal as a temporal variation in measured SOC over a defined time interval and the noise as the corresponding spatial prediction error. Although the specific definitions and calculations of SNR differ across studies, our findings are consistent with those of Broeg et al. (2024). In both cases, SNR values rarely exceed one over short time spans, surpassing this threshold only after approximately 25 years of observation, and SNR is higher for measurements with higher SOC contents (> 15 g/kg). This convergence underscores the robustness of SNR as a diagnostic metric and the importance of long‐term monitoring for capturing SOC change.

Large‐scale SOC change assessments are often based on prediction time series using the *state‐first* approach, as repeated measurements are typically unavailable. In such contexts, SNR provides a practical and interpretable means of quantifying the detectability of predicted changes when direct validation is infeasible. It can therefore serve as a readily available indicator of the reliability of SOC change estimates in widely used SOC mapping products. When repeated measurements are available, *change‐first* approaches become feasible, allowing overall error metrics to be computed by directly comparing predicted and observed changes. However, these metrics remain global summaries and are limited to locations where measurements exist. By contrast, because SNR can be computed for every individual prediction, it can function as a spatially explicit quality indicator across the full prediction domain.

In this sense, SNR complements traditional accuracy metrics by providing additional insight into the reliability and interpretability of modeled SOC change, but it should not be viewed as a replacement for predictive accuracy. Accuracy metrics evaluate model performance against observations, whereas SNR serves as an internal diagnostic that indicates whether predicted changes exceed the model's own uncertainty. A key prerequisite for meaningful SNR assessment is valid uncertainty estimation. In this study, all three models—$RF_{c}$, $RF_{\delta }$, and $RF_{\beta }$—produced reasonably calibrated uncertainty intervals, as demonstrated by both coverage (Figure 4) and sharpness (Supplementary Notebook 15). Under this condition, SNR provides information on whether the model itself considers a predicted change to be distinguishable from its associated uncertainty, independent of whether the prediction is ultimately accurate.

We further compared SNR with predictive accuracy at the point level (Supplementary Notebook 12). Only weak correlations were observed between SNR and relative absolute error (Spearman's ρ=0.17) for both $\hat{\delta }$ and $\hat{\beta }$. At first glance, this weak relationship, together with the generally poor predictive accuracy, may raise questions about the validity of the proposed SNR framework. However, across all modeling strategies, we consistently observe both low predictive accuracy and low SNR. These outcomes are mutually consistent, indicating that SOC change signals are dominated by noise and therefore offer limited interpretive value. As soil monitoring networks such as LUCAS expand, national soil surveys continue, and EO and ancillary datasets improve, longer and denser time series are expected to support more robust models and higher signal‐to‐noise ratios. Under such conditions, SNR may align more closely with external accuracy measures and provide a richer basis for investigating where, why, and under which conditions SOC change becomes more detectable.

### 4.3 Factors Influencing SNR

When comparing SNR between $\hat{\delta }$ and $\hat{\beta }$ for the same time span, we observed higher SNR values for $\hat{\beta }$, which incorporates a third SOC observation. In contrast, signal, noise, and SNR of δ remained largely stable despite increasing intervals between paired two observations (Table 5). This highlights the importance of not only long‐term monitoring, but also adequate temporal sampling density. Using data from Denmark's long‐term Soil Monitoring Network, Harbo et al. (2023) showed that short‐term fluctuations can mask long‐term SOC trends, making it risky to estimate δ from only two observations. Similar short‐term fluctuations are also evident in the LUCAS repeated measurements Supplementary Notebook 11b, which can be partially smoothed during trend fitting when multiple time steps are available. Consistently, Broeg et al. (2024) showed that SNR increases with longer observation periods. The availability of LUCAS 2022 data will allow further assessment of this effect.

Compared to the *state‐first* approach, *change‐first* models generally produced more variable SNR values across land‐cover series for both δ and β. While both approaches yield variation in predicted changes across land‐cover contexts, the *change‐first* approach exhibits more stable uncertainty estimates across land‐cover types, suggesting the presence of a baseline noise level in its predictions. As a result, differences in SNR across land‐cover series are more strongly driven by variation in the predicted signal than by changes in uncertainty.

For specific land‐cover contexts—such as *Woodland* for δ (Figure 6) and both *Woodland* and *Grassland* for β (Figure 7)—the *change‐first* approach generally yields higher SNR values than the *state‐first* approach, whereas lower SNR values are observed for other land‐cover settings. Notably, these patterns appear to be more closely related to the presence of *Woodland* or *Grassland* in the land‐cover history than to the occurrence of land‐cover transitions themselves. For example, sites classified as *Grassland* throughout the entire observation period show higher SNR values than sites experiencing *Grassland–Cropland* or *Cropland–Grassland* transitions (Figure 7), despite the latter being expected to exhibit larger SOC changes. Although increasing vegetation cover can mask soil signals and reduce detectability from a sensing perspective, dense vegetation is often associated with higher SOC levels, which may counterbalance this effect. This observation is consistent with the findings of Broeg et al. (2024), who reported that higher SNR is associated with larger background SOC signals, which are commonly found in *Woodland* and *Grassland* systems.

### 4.4 Spatial Aggregation Improves SNR, but Not Always

The results confirm that spatial aggregation can improve SNR (Figure 9). For δ, noise decreased more rapidly than signal with increasing aggregation size, resulting in a clear increase in SNR. The reduction in noise is primarily a statistical effect: when aggregating over larger spatial units, random and spatially uncorrelated errors tend to cancel out (Smith 2004; Wadoux and Heuvelink 2023, 2025). In contrast, the aggregated signal becomes smaller and less variable as it is dominated by the prevalence of small or near‐zero changes within the aggregation unit. For β, both signal and noise decrease with increasing aggregation size at relatively similar rates, leading to largely stable SNR values, with only a slight increase at the largest aggregation scale (200 km^2^). This difference likely reflects the fact that β is estimated from multiple time steps, which already suppresses part of the short‐term variability and measurement noise during the trend‐fitting stage. As a result, spatial aggregation provides less additional noise reduction for $\hat{\beta }$ than for $\hat{\delta }$. Despite the weaker aggregation effect, $\hat{\beta }$ generally exhibits higher SNR values than $\hat{\delta }$, indicating greater detectability of trend‐based change estimates across spatial scales.

Another factor influencing SNR behavior is the spatial domain being mapped—specifically, its environmental conditions, the underlying pattern of pixel‐level SNR, and the spatial structure of model errors. In our study, the improvement was more pronounced in Spain (Figures 9 and 10). Although SNR also increased with aggregation in Germany, the improvement was smaller and insufficient to exceed one, even at 200 km^2^ support. This contrast primarily reflects environmental and land‐cover differences: Spain's more sparsely vegetated landscapes produced smaller signal magnitudes but also lower uncertainties than Germany (Figures 6 and 7). Furthermore, the longer variogram range observed in Germany suggests that spatially correlated errors persist over larger distances, limiting the benefits of aggregation (Wadoux and Heuvelink 2023).

The choice of aggregation size and unit also influences SNR behavior through the Modifiable Areal Unit Problem (MAUP) (Openshaw and Taylor 1979; Dark and Bram 2007). The results revealed the scale effect of the MAUP, where analytical outcomes vary with aggregation level. While SNR generally increased with coarser resolution, the trend was not strictly monotonic—for instance, in parts of northeastern Spain, SNR rose at 40 km^2^ but declined again at 200 km^2^ as low‐SNR areas became dominant within the larger grids. The zonal effect of MAUP—where different zoning schemes (e.g., aggregation by regular grids or administrative units) yield distinct spatial patterns—also needs consideration, although it was not examined in this study. When pixel‐level SNR is homogeneous within a unit, aggregation reduces noise and preserves the signal, thereby improving SNR, as observed in parts of northwestern and central Spain. In contrast, in heterogeneous areas, strong local signals may be masked by surrounding low‐SNR regions, limiting the benefits of aggregation.

Aggregation strategies should also consider their practical relevance for MRV, which depends on contextual factors such as land use, management practices, and local regulations that vary spatially (Smith 2008; Gocke et al. 2023). For instance, management may differ considerably between neighboring farms, yet in some areas, practices are relatively homogeneous due to shared traditions or policy requirements. In this study, we used regular grids for demonstration purposes, but in practice, aggregation units should reflect local land‐use and management contexts. For example, in the Netherlands, farmers are legally required to grow cover crops after maize cultivation (Fan et al. 2020), resulting in relatively uniform management across large areas. In such cases, aggregation at administrative or farm levels may be more meaningful than uniform grid cells.

However, this improvement comes with a trade‐off: increasing aggregation enhances detectability but reduces spatial detail and local interpretability. Although higher SNR with aggregation is statistically expected, it does not necessarily imply improved model skill. Depending on the aggregation scheme, aggregation may yield little benefit or lead to apparent improvements less meaningful or representative. Ultimately, incorporating domain knowledge, local context, and intended use into aggregation design is necessary to ensure the interpretability and policy relevance of SOC change assessments.

### 4.5 Strengths and Challenges of Using LUCAS for SOC Change Modeling

LUCAS forms the cornerstone of this study. As of this writing, three survey rounds (2009/2012, 2015, and 2018) are publicly available, covering a total of 9 years—still a relatively short period for reliable modeling of SOC change using ML and EO data alone. In Denmark, decadal sampling has been suggested as sufficient to capture long‐term SOC trends, as short‐term fluctuations may not reflect meaningful change (Harbo et al. 2023). The upcoming release of LUCAS 2022 will extend the record beyond a decade and provide an additional time step for trend analysis, increasing the potential for detecting long‐term SOC dynamics.

While LUCAS is an exceptional resource, several inherent characteristics limit its suitability for SOC change modeling. Designed to represent all of Europe with roughly 20,000 sites (Orgiazzi et al. 2018), it effectively captures broad spatial patterns. However, this continental scope can mask subtle local or temporal variations in SOC, particularly in large‐scale mapping frameworks where a single generalized model is applied across all locations and time steps (Lemercier et al. 2022). In addition, the relatively low sampling density of such continental surveys restricts their suitability for fine‐scale spatial aggregation, as fitting spatial variograms requires sufficient site density at short distances. For this reason, denser national surveys were used in this study to derive standardized error variograms for the spatial aggregation analysis.

Coordinating sampling across many member states and survey years also poses challenges for maintaining consistency. Although all samples are analyzed using standardized methods, variations in both laboratory and field implementations may still introduce non‐negligible differences—particularly given the subtle SOC changes being monitored. Laboratory‐related inconsistencies, for example, have been reported in alkaline soils, where higher analytical uncertainties have been observed in SOC measurements (Schneider et al. 2021). Field‐related inconsistencies are also reflected in the extreme apparent SOC changes observed (Figure 3). In some cases, δ reaches 400 g/kg and β up to 50 g/kg/year—values that far exceed realistic expectations (Poeplau and Don 2013; Gubler et al. 2019). Many of these outliers occur in *Woodland* areas, where inconsistent separation of organic litter from mineral soil layers is suspected (Hiederer 2020; Ziche et al. 2022). Another likely source of such anomalies is the destructive nature of soil sampling: exact resampling of the same point is impossible. Although LUCAS mitigates this through composite sampling, the official data evaluation report (Hiederer 2020) emphasizes that repeated samples should be interpreted as representative of a small area rather than a fixed point. Minor spatial displacement between survey rounds can therefore generate apparent SOC differences unrelated to temporal change, particularly where spatial heterogeneity exceeds temporal variability (Poeplau et al. 2022; Broeg et al. 2024).

Another source of uncertainty lies in the sampling method itself. In LUCAS, soils are sampled with a spade. While this approach facilitates continent‐wide soil surveys at relatively low cost, it raises concerns about whether spade‐based samples accurately represent the upper 0–20 cm layer in certain land uses. Comparative studies from Switzerland indicate that hand‐auger sampling provides greater precision, particularly in *Grassland* and forest soils with pronounced SOC depth variation (Fernández‐Ugalde et al. 2020). Furthermore, repeated LUCAS soil measurements are currently limited to the 0–20 cm topsoil layer. Although this is where most SOC change typically occurs, management effects in croplands have been reported to extend to depths of up to 50 cm (Skadell et al. 2023).

Several refinements could further enhance LUCAS's capacity for SOC change modeling and monitoring. Permanent site marking or more detailed and standardized location referencing could help reduce relocation uncertainty. Likewise, replacing spade‐based sampling with hand augers would improve depth control and the representativeness of samples within the 0–20 cm layer. LUCAS already provides detailed survey‐level quality reports—an excellent practice that promotes transparency. Extending this to the individual record level, for example by including per‐sample quality flags (analogous to pixel‐level quality indicators in remote‐sensing datasets), would further enhance data consistency and interoperability. National soil surveys—such as those from Spain and Germany used here—complement LUCAS by adding spatial detail and sampling depth, and stronger coordination with national monitoring networks and EO datasets could further improve temporal consistency and model robustness (Froger et al. 2024). While some refinements could increase precision, they would also raise costs. Therefore, we recommend prioritizing denser and more frequent resampling of existing LUCAS sites to build consistent long‐term time series, supported by improved and standardized metadata for uncertainty assessment.

Taken together, these recommendations are not criticisms of LUCAS but reflect the challenges of applying a continental‐scale survey to high‐resolution SOC change modeling. LUCAS was designed to provide harmonized, long‐term environmental data rather than detailed modeling inputs and already achieves a strong balance between spatial coverage, cost, and feasibility. The program has continuously evolved based on lessons from previous rounds, and the recent inclusion of subsurface layers (e.g., 20–30 cm) marks a valuable step toward capturing vertical SOC trends. Its continuation remains vital for SOC dynamics modeling and will further strengthen the empirical basis for model calibration and improve SNR.

### 4.6 Implications for SOC Monitoring and DSM Applications

Our findings support the conclusions of Broeg et al. (2024): reliable SOC change detection at fine spatial resolutions (e.g., point, site, or pixel level) remains difficult when relying on EO data and ML methods alone. High prediction uncertainty, limited time‐series length, and the inherently slow dynamics of SOC all contribute to this limitation. Making these constraints explicit and measurable is a necessary step toward more transparent interpretation of DSM‐based SOC change products. In this context, SNR provides a quantitative diagnostic by explicitly relating predicted change magnitude to its associated uncertainty. Low‐SNR values should therefore be interpreted as a warning against over‐interpretation of modeled SOC change, indicating that predictions are likely dominated by noise. SNR can nevertheless be used in spatial aggregation analyses, where aggregation may improve detectability. In addition, spatial patterns of SNR help identify regions with relatively higher or lower detectability, and locations or aggregation units with higher SNR may serve as starting points for investigating environmental or land‐use conditions associated with more detectable SOC change signals.

Improving SOC change detectability will depend heavily on the quality of data. To achieve higher SNR, longer, consistent observational datasets—like those from the LUCAS program—are needed to improve the training, validation, and understanding of SOC change models. Beyond extending temporal depth or increasing spatial support, additional strategies can improve SNR, including the use of more relevant, higher‐quality covariates and advances in model architectures (Heuvelink et al. 2021; Tian, de Bruin, et al. 2025). Hybrid approaches that combine the strengths of ML models and domain‐specific process knowledge have emerged as a promising direction (Reichstein et al. 2019). Notable examples include constraining numerical models with domain knowledge (De Rosa et al. 2024), coupling process‐based and ML models through loss functions (Zhang, Heuvelink, et al. 2024), replacing sub‐modules of complex process‐based models with ML components (Liu et al. 2024), and augmenting limited temporal SOC datasets with synthetic data generated by process‐based models (Zhang, Heuvelink, et al. 2024).

That said, time series of high‐resolution DSM products remain valuable. In fact, $\hat{c}$ often yield SNR larger than one, demonstrating their value in capturing spatial SOC patterns. Moreover, this SNR evaluation framework can be adapted to work with typical DSM outputs. These predictions can also be aggregated to coarser spatial units, where the resulting SOC changes tend to exhibit higher SNR, indicating more certain and interpretable trends. Given the limited availability of repeated SOC measurements, especially in regions without long‐term monitoring programs like LUCAS, the *state‐first* approach will likely remain the most viable strategy for detecting and reporting SOC change in the near future. As long as corresponding SNR values are reported alongside SOC change estimates, this approach still support reliable interpretation of model outputs and help identify areas of meaningful change. We advocate for more inclusion of SNR metrics in SOC change estimate efforts to better account for uncertainty and guide data‐informed decisions.

### 4.7 Limitations

The SNR analysis framework developed in this study evaluates the reliability of SOC dynamics modeling by comparing predicted SOC change magnitudes (signal) against model‐derived uncertainty (noise). Since both components are produced by the ML model, the validity of the resulting SNR values depends on the accuracy of both the predictions and the associated uncertainty estimates. In this study, we employed RF and QRF, which are commonly used in ML‐based DSM to generate predictions with uncertainty (Wadoux et al. 2020). While we expect our conclusions to hold for other models with robust uncertainty quantification, further work is needed to confirm their generalizability.

Additionally, while we included the trend‐based metric β in our analysis, it was estimated from only three SOC measurements over time. Such limited temporal depth introduces uncertainty in trend estimation. Given current data availability, this remains the most feasible approach. However, the forthcoming LUCAS 2022 dataset is expected to provide an additional time step, enabling more reliable trend estimates and reinforcing the findings of this study.

Although predicted changes inherently contain both magnitude and direction, the SNR metric, as implemented in this study, quantifies magnitude‐based detectability. With respect to direction, under the assumption of symmetric prediction uncertainty, a sufficient but not necessary condition for directional inference is an SNR value exceeding 0.5, which implies that the prediction distribution lies entirely on one side of zero change. In this study, however, SNR values remain below this level for the vast majority of predictions, substantially limiting the practical relevance of directional inference under current data availability. When the conditional prediction distribution is asymmetric, directionality cannot be inferred from a single summary measure of uncertainty; upper and lower uncertainty bounds must be considered separately. Under such conditions, change direction may still be resolvable even when overall SNR remains low. While the direction of change is an important aspect of SOC dynamics, its reliable assessment requires a dedicated methodological framework, potentially involving classification‐based models and explicit treatment of directional uncertainty, which lies beyond the scope of the present study and could be addressed in future work.

While this study provides a quantitative demonstration of how spatial aggregation can improve the SNR, the analysis was intentionally simplified to illustrate the concept rather than to exhaustively evaluate all possible aggregation scenarios. The results should therefore be viewed as a demonstration of the general potential to identify SOC trends more reliably at larger spatial scales, rather than as an attempt to quantify national or regional SOC changes (e.g., in Germany or Spain). Land use and management—key drivers of SOC dynamics—were not explicitly considered in this analysis due to the lack of harmonized, temporally resolved management data at the continental scale. Land‐use data at large scales, although increasingly available through ML‐based EO products, remain subject to uncertainty—just like to that of SOC maps derived from DSM. Data on management practices are even scarcer: existing datasets are typically static in time (Sandström et al. 2023) or available only at coarse spatial resolution (Lampach et al. 2025). The omission of these factors means that part of the observed noise, particularly at the point scale, may reflect unaccounted management heterogeneity. Moreover, the spatial aggregation analysis was restricted to the *state‐first* approach using the $RF_{c}$ model. Extending the framework to $RF_{\delta }$ or $RF_{\beta }$ models would in principle be possible, but would require denser temporal datasets to support reliable variogram fitting—data that are not yet available.

Future work should aim to integrate contextual information—such as land use and management practices—into both SOC dynamic modeling and spatial aggregation analyses. Incorporating these factors would strengthen the mechanistic understanding of SOC change and enhance the policy relevance of results. Further research should also systematically evaluate how different aggregation schemes—varying in both scale and zoning design—affect SNR behavior across contexts (e.g., farm management, policy reporting, and global monitoring). Such analyses would help clarify the mechanisms by which aggregation enhances or diminishes detectability and provide empirical guidance for designing effective MRV systems. Without detailed land‐use and management information or a systematic assessment of aggregation effects, the interpretability of aggregated SNR values—and thus their usefulness for understanding underlying SOC processes—remains limited.

## 5 Conclusions

With SNR consistently below one and low prediction accuracy, our study demonstrates that, under current data availability, modeling SOC dynamics at the site level using ML and EO data alone remains infeasible. The limited number and frequency of repeated SOC observations constrain the ability to capture meaningful temporal trends. Both the *state‐first* and *change‐first* approaches exhibit similarly poor predictive performance, although the *change‐first* method provides more consistent yet typically narrower uncertainty estimates. While a longer monitoring period does not necessarily increase SNR, incorporating more time steps into SOC change quantification tends to improve it. Higher SNR values are also observed in land covers with greater vegetation content, such as *Woodland* and *Grassland*.

SNR, as an internal metric for assessing the detectability of modeled SOC changes, cannot replace independent validation. It offers a valuable indicator of change detectability, given the scarcity of repeated SOC observations and can be applied to evaluate the prevailing practice of mapping SOC dynamics through the *state‐first* approach. When repeated SOC measurements become available, SNR could complement traditional performance metrics by providing spatially explicit insights into the reliability of modeled SOC changes. We argue that SNR should be routinely reported when interpreting or claiming SOC change from model predictions. Without explicitly accounting for uncertainty, such maps risk overstating their reliability for MRV purposes.

Finally, our analysis confirms that spatial aggregation improves SNR, with the degree of improvement depending on both the spatial structure of model predictions and the choice of aggregation units. This suggests that, while site‐level assessments remain challenging, reliable SOC change modeling is still achievable at larger scales. This offers a promising pathway for regional‐scale monitoring and reporting. Nonetheless, careful selection of aggregation scales and units is required to ensure that reported SNR values remain meaningful and representative.

## Author Contributions

**Xuemeng Tian:** conceptualization, methodology, software, data curation, validation, writing – review and editing, visualization, investigation, writing – original draft, formal analysis. **Sytze de Bruin:** conceptualization, writing – review and editing, supervision, investigation, visualization, formal analysis, methodology. **Florian Schneider:** conceptualization, writing – review and editing, writing – original draft, supervision, investigation, data curation. **Martin Herold:** conceptualization, writing – review and editing, supervision. **Kirsten de Beurs:** conceptualization, writing – review and editing, supervision.

## Conflicts of Interest

The authors declare no conflicts of interest.

## Data Availability

The data and code supporting the findings of this study are openly available via Zenodo at https://doi.org/10.5281/zenodo.18706448. Soil data from Germany (BZE‐LW) are openly available at https://doi.org/10.3220/DATA20200203151139. Soil data from Spain (Parcelas COS and Parcelas INES) are openly available at https://www.miteco.gob.es/content/dam/miteco/es/biodiversidad/servicios/banco‐datos‐naturaleza/2‐cos/bbdd‐cos.zip. LUCAS Soil Survey data are available from the European Soil Data Centre (ESDAC) for the respective survey years: 2009 and 2012 at https://esdac.jrc.ec.europa.eu/content/lucas‐2009‐topsoil‐data, 2015 at https://esdac.jrc.ec.europa.eu/content/lucas2015‐topsoil‐data, and 2018 at https://esdac.jrc.ec.europa.eu/content/lucas‐2018‐topsoil‐data.
